# Fatty fish intake and attention performance in 14–15 year old adolescents: FINS-TEENS - a randomized controlled trial

**DOI:** 10.1186/s12937-017-0287-9

**Published:** 2017-10-02

**Authors:** Katina Handeland, Jannike Øyen, Siv Skotheim, Ingvild E. Graff, Valborg Baste, Marian Kjellevold, Livar Frøyland, Øyvind Lie, Lisbeth Dahl, Kjell M. Stormark

**Affiliations:** 1 National Institute of Nutrition and Seafood Research (NIFES), P.O.Box 2029 Nordnes, 5817 Bergen, Norway; 2 Department of Clinical Medicine, Faculty of Medicine, University of Bergen, P.O. Box 7800, 5020 Bergen, Norway; 3 Regional Centre for Child and Youth Mental Health, Uni Research Health, P.O.Box 7810, 5020 Bergen, Norway; 4 Uni Research Health, P.O.Box 7810, 5020 Bergen, Norway; 5 Department of Health Promotion and Development, University of Bergen, P.O.Box 7807, N-5020 Bergen, Norway

**Keywords:** Fatty fish, Omega-3 fatty acids, Meat, Cognition, Supplements, Food, Dietary intervention

## Abstract

**Background:**

Fatty fish is the dominant dietary source of n-3 LCPUFAs but it also contains other micronutrients considered important for brain development and function. To our knowledge, the effect of fatty fish intake on cognitive function in adolescents has not been investigated in randomized controlled trials (RCTs) previously. The aim of the present trial was to investigate whether consumption of fatty fish meals three times per week for 12 weeks could alter attention performance in adolescents compared to similar meals with meat or n-3 LCPUFA supplements.

**Methods:**

In the Fish Intervention Studies-TEENS (FINS-TEENS), adolescents from eight secondary schools (*n* = 426; age: 14-15y) were individually randomized. Attention performance was assessed with the d2 test of attention. Differences between groups from pre to post intervention were assessed with linear mixed effect models and general estimates equation. The fish group was set as reference. Dietary compliance was recorded for each meal throughout the trial and controlled for in the adjusted analyses.

**Results:**

The improvement in processing speed was significantly lower in the meat (−11.8; 95% CI: -23.3, −0.4) and supplement (−13.4; 95% CI: -24.9, −1.8) group compared to the fish group (reference). The supplement group also showed inferior improvement in total performance (−10.4; 95% CI: -20.0, −0.7) compared to the fish group (reference). The results were slightly affected when controlling for dietary compliance. Omission errors decreased in the meat group compared to the fish group (Incidence rate ratio = 0.85; 95% CI: 0.74, 0.98), but the difference disappeared when controlling for dietary compliance.

**Conclusions:**

We observed a small beneficial effect of fatty fish, compared to meat meals and supplements on processing speed. However, these results are difficult to interpret due to low dietary compliance. This study shows that different taste preferences among participants is challenging in intervention trials with food. A prospective cohort design may be a better alternative when studying diet in the future.

**Trial registration number:**

ClinicalTrials.gov registration number: NCT02350322.

**Electronic supplementary material:**

The online version of this article (10.1186/s12937-017-0287-9) contains supplementary material, which is available to authorized users.

## Background

Subtle alterations in the diet have the potential to influence cognitive function in humans, even in industrialized countries where the food-availability is good [1]. Most studies have focused on the role of nutrition in early childhood when brain development is at its peak and particularly sensitive to insults due to dietary deficiencies [2]. However, the brain continues to develop during adolescence, and relatively little is known about the role of nutrition on cognitive function in adolescents from mainstream school populations [3].

A growing body of evidence suggests that an adequate intake of the omega-3 (n-3) long-chain polyunsaturated fatty acids (LCPUFAs) is of importance for brain development and function [4]. This association is plausible, since eicosapentaenoic acid (EPA) and docosahexaenoic acid (DHA) are important structural components of neural cell membranes. They influence the brain through membrane fluidity, myelination and neurotransmission [5, 6]. In addition, the dietary intake of n-6 fatty acids is in excess in the European population, introducing a potential imbalance between the n-6 and n-3 PUFAs in the body [7, 8]. However, meta-analysis of randomized controlled trials (RCTs) have not found evidence that n-3 PUFA supplementation impacts cognition in healthy subjects [9, 10]. The results are also inconclusive in healthy adolescents, and two independent RCTs found no benefit of supplementing with DHA alone or DHA + EPA on cognitive performance in schoolchildren [11, 12].

Fish consumption in adolescents has been associated with better school achievements and performance in cognitive tests [13–15]. One RCT found improved reading performance after a school meal intervention including fish approximately once a week [16]. Fatty fish like mackerel, herring and salmon is the dominant dietary source of n-3 LCPUFAs, but these species also contain other micronutrients considered important for the brain, e.g. vitamin D, iodine, and vitamin B_12_ [1, 17]. The diet of Norwegian adolescents contains little fish [18]. Thus, it is reasonable to hypothesize that an increased intake of fatty fish could be more beneficial compared to an intake of meat, and potentially also compared to an intake of supplements with n-3 LCPUFAs. To our knowledge, no RCTs have previously addressed the specific impact of eating fish on cognition in adolescents.

The aim of the present RCT was to investigate whether fatty fish meals three times per week for 12 weeks altered attention performance in adolescents (14–15 y), compared to similar meals with meat or supplements with n-3 LCPUFAs.

## Methods

### Study design and ethics

Fish Intervention Studies-TEENS (FINS-TEENS) was conducted at eight lower secondary schools in Bergen, Norway, between February and May 2015. The three-armed trial used a RCT design to investigate the cognitive effects of providing meals with fatty fish or similar meals with meat/cheese or fish oil supplements to adolescents from a mainstream school population.

The trial was conducted according to the declaration of Helsinki. Ethical approval was obtained from the Norwegian Data Protection Official for Research (project number: 41030). Written informed consent was collected from all participants and one parent/caregiver, and participants could withdraw from the trial without giving any reason. The trial is registered in ClinicalTrials.gov (NCT02350322). The design and methods are described in detail in Skotheim et al. [19].

### Subjects and randomization

Inclusion criteria were that the adolescents were attending 9^th^ grade at the schools participating in the study, and that they knew the Norwegian language orally and written. Thus, all pupils at 9^th^ grade received invitation to participate in the study. Exclusion criteria were allergy or intolerance to the study food or supplements. The random allocation was performed individually stratified by sex. Two researchers, (one blinded), assigned every enrolled girl and boy to either the fish, meat or supplement group by drawing lots. Researchers and participants were not blinded to treatment conditions in the present study, but the d2 test of attention was scored blinded.

### Dietary intervention procedure

The meals and supplements were delivered to participants at school during their habitual lunch break (usually between 11:00 a.m. and noon). The participants ate together in their respective classrooms. The fish- and meat meals replaced the participants’ habitual school lunch, whereas participants in the supplement group continued to eat their habitual lunch in addition to consume the supplements. Participants received the meals or supplements three times per week for 12 weeks. The school lunch in Norway usually consists of a packed lunch from home, containing medium dark or dark bread or crispbread with meat, cheese or liver pate as spread, and sometimes a fruit or vegetable [18, 20].

The fish- and meat meals were prepared by catering service. Meals in the fish group contained salmon, mackerel and herring, whereas the meals in the meat group contained chicken, turkey, beef, lamb and cheese. Halal meat was provided on request and pork meat was not used in the trial. The meals were similar in content except from the meat and fish, and contained vegetables and/or salad and mainly wholegrain pasta-, focaccia-, baguette- or tortilla and sometimes dressing. Gluten free products were provided on request for gluten sensitive participants, but those with celiac disease were excluded from the trial because 100% gluten-free meals could not be guaranteed. The weekly content of n-3 LCPUFAs was matched between the supplement and fish group by using a mean of 90 g fish per serving as reference to determine the number of capsules, which were seven per serving. The supplements were bought at a public pharmacy and each capsule contained 500 mg of concentrated fish oil, of which 158 mg was EPA, 105 mg was DHA and 13 mg was docosapentaenoic acid (DPA) (Nycoplus® Omega-3, 500 mg, Takeda Nycomed, Asker, Norway). The meals had a mean weight of 230 g/portion and the meals in the fish group contained on average 2.1 μg/100 g of vitamin D, 4.9 μg/100 g of iodine, 152.3 mg/100 g EPA, 262.3 mg/100 g DHA and 39.9 mg/100 g DPA. The meals in the meat group contained on average < 1 μg/100 g of vitamin D, 2.6 μg/100 g of iodine, 3.2 mg/100 g of EPA, 5.0 mg/100 g DHA and 6.0 mg/100 g DPA.

Dietary compliance was recorded for each participant throughout the trial by study staff. They counted the remaining capsules and estimated by eye the amount of fish/meat eaten using a scale from zero to four: ‘0 = nothing eaten’, ‘1 = 1/4 eaten’, ‘2 = 2/4 eaten’, ‘3 = 3/4 eaten’ and ‘4 = all eaten’.

### Outcomes

#### Cognitive tests

The primary outcome was attention performance measured by the d2 test of attention [21]. In addition, performance in a Norwegian reading and spelling test named “Kartleggeren” was assessed but not reported, due to considerable ceiling effects in nearly all outcomes at pre and post intervention. Mental health status was measured with the Strengths and difficulties questionnaire (SDQ) and reported in a separate publication (Skotheim et al. [19]). Thus, only results from the d2 test of attention are presented in the present paper.

The d2 test of attention is a pen and paper attention cancellation test. The subjects’ task is to cancel out as many target characters (a “d” with a total of two dashes above and/or below) as possible, while they ignore distraction characters (“d’s” with more or less than two dashes and “p’s” with any number of dashes) [21]. The test comprises 47 interspersed target- and distraction characters × 14 rows. It is conducted under time pressure, as participants are only allowed 20 s on each row before they must move down to the next, independent of how far they reach. The test, including instructions, was administered within 8 min. The test has shown to be a concise and valid measure of selective attention and mental concentration, comprising measures of visual scanning, processing speed and degree of accuracy, regardless of intelligence level [21, 22].

The test outcomes are described in Table 1, and were ‘concentration performance’ (CP), ‘total performance’ (TN-E), ‘processing speed’ (TN), ‘omission errors’ (E1) ‘commission errors’ (E2) and ‘total errors’ (Table 1).

**Table 1:** Overview and description of the different outcomes of the d2 test of attention

Outcome	Description
Concentration performance (CP)	Total number of correctly cancelled out target characters minus commission errors
Total performance (TN-E)	Total number of characters processed minus total errors made
Processing speed (TN)	Total number of characters processed
Omission errors (E1)	Unmarked target characters
Commission errors (E2)	Incorrectly marked distraction characters
Total errors	The sum of E1 and E2 errors

The cognitive testing was conducted in classrooms under controlled conditions. The same study crew administered the test at pre and post at approximately similar timepoints (between 09:00–11:00 a.m.). Classroom noise was rigorously controlled for by the study crew and the teachers, and trained research staff introduced the test according to standard instructions.

#### Questionnaire

Participant characteristics (age, weight, height and sex) and background diet (habitual dietary intake besides the intervention) was obtained with a revised and extended version of a validated web-based food frequency questionnaire (FFQ) at pre and post intervention [23, 24]. Body mass index (BMI) was calculated as weight in kilograms divided by the square of the height in meters (kg/m^2^). To classify weight status, Cole’s age and sex-specific BMI cut off points for underweight [25], and overweight and obesity [26] for adolescents (14.5 years) were used. The cut offs for thinness are 16.7 for boys and 17.2 for girls. For overweight the cut offs are 23.0 and 23.7 for boys and girls respectively, and for obesity they are 28.0 and 28.9 for boys and girls, respectively. A questionnaire was sent to the parents/caregivers by e-mail, and assessed their level of education, total household income and origin. The participants were requested to maintain their daily routines (dietary pattern, use of dietary supplements, physical activity level, etc.) for the duration of the trial.

#### Blood sampling and biochemical analyses

Blood and urine samples were also collected to measure the status of nutritional biomarkers from pre to post intervention. These data will be presented in a separate publication.

### Sample size

The sample size calculation was based on the design with a three armed-intervention, with two repeated measurements (pre and post intervention) with an assumed correlation of 0.5. To be able to reveal a meaningful effect of the intervention, a small to moderate effect size (0.35) on the main outcome (d2 test of attention) was applied. It was calculated that a sample size of 119 participants was needed in each group given a power of 80% and a significance level of α = 0.05. Taken into account a 20% drop out rate, the aim was to enrol a total sample of 446 participants in the trial. The sample size calculation was carried out using Stata Statistical Software: Release 14. College Station, TX: (STATACorp LP®).

### Statistical analyses

Continuous data are presented as mean and standard deviations (SD) and categorical data are presented as number (n) and percent (%). Differences between intervention groups in baseline data was assessed with one-way ANOVA (continuous variables) or Chi-square test (categorical variables). Differences between drop-outs and completers were assessed with independent samples t-test (continuous variables) or Chi-square test (categorical variables).

The analyses were carried out on all participants for whom pre and post data from the d2 test of attention were available. Differences in pre to post intervention in the d2 test outcomes within intervention groups were assessed with paired samples t-test.

To estimate the differences between intervention groups in change from pre to post intervention, linear mixed effect models were applied for the normally distributed outcomes (CP, TN-E, TN). The participants’ school class was included as random intercept to account for dependency in the data at the level of class affiliation. The error-outcomes from the d2 test followed a negative binomial distribution, and were analyzed using generalized estimates equation (GEE) model with exchangeable correlation structure and robust standard errors. The GEE model account for the nested design of children clustered within class, and estimated incidence rate ratio (IRR) with 95% confidence interval (CI) were presented. For each outcome, two models were presented. In both models, the dependent variable was the currently examined d2 outcome at post intervention, adjusted for the equivalent outcome at baseline. The second model additionally included dietary compliance. The fish group was used as reference. Model assumptions were investigated by visual inspections of residual- and normal probability plots.

Additional analyses with further adjustment for parental education level, at home omega-3 supplement use and fatty fish intake (dinner and bread spread) at baseline were performed. In addition, an interaction between intervention and dietary compliance were included in the model.

Pearson correlations were performed between dietary compliance and the change (post-pre) in TN and TN-E (normally distributed outcomes), and Spearman correlation was performed between dietary compliance and change in E1 errors, for each intervention group.

Two-tailed *p* values <0.05 were considered statistically significant. Statistical analyses were carried out using the Statistical Package for the Social Sciences (SPSS® Statistics version 24, IBM Corporation, US), except for the linear mixed effect models and GEE, which were performed using Stata Statistical Software: Release 14. College Station, TX: (STATACorp LP®).

## Results

### Subjects

**Fig. 1:**
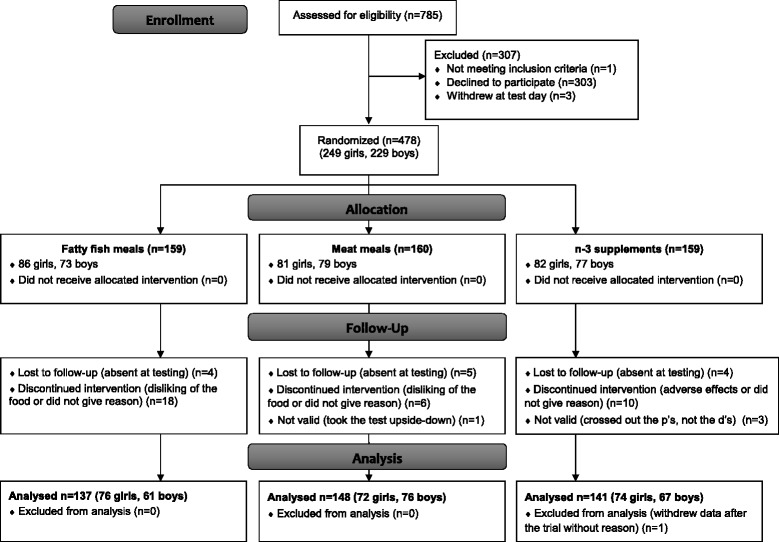
Flow chart over participants. n-3 = omega-3

Of the 785 adolescents who received invitation to participate, 303 declined the invitation and one did not meet inclusion criteria. Thus, 481 were enrolled in the trial. Three participants withdrew at the day of baseline testing before randomization. As a result, 478 participants were randomized. During the trial, 34 withdrew mainly because they disliked the intervention food or the supplements, or without giving any reason. In addition, 17 participants were lost to follow up during testing at pre or post intervention, and one participant withdrew his consent after study completion. Thus, pre and post data from the d2 test of attention were available for 426 (86%) participants, on which the final data analyses were conducted (Fig. 1). Drop-outs and participants lost to follow up did not differ from completers in any of the baseline characteristics (data not shown).

Baseline characteristics of the participants are summarized in Table 2. Participants had a mean age of 14.6 ± 0.3 years and 81% had a BMI within the normal range. Almost all participants were non-immigrants, defined as both themselves and their parents were born in Norway. Approximately 30% of the parents/caregivers had a high family income (>1,250,000 mill. Norwegian kroners (which is equivalent to approximately $144,000), and 61% had college/university degree. Intervention groups did not differ with regard to baseline characteristics or baseline dietary intake of fish (data not shown).

**Table 2:** Baseline characteristics of all participants and by intervention groups

Variables	N	All (*n* = 426)	Fish (*n* = 137)	Meat (*n* = 148)	Supplement (*n* = 141)	*P-value* ^*a*^
Sex n (%)	426					0.512
Male		204 (47.9)	61 (44.5)	76 (51.4)	67 (47.5)	
Female		222 (52.1)	76 (55.5	72 (51.4)	74 (52.5)	
Age (years) mean ± SD))	426	14.6 ± 0.3	14.6 ± 0.3	14.6 ± 0.3	14.6 ± 0.3	0.718
BMI category^b^ (kg/m^2^)	396					0.250
Underweight n (%)		53 (12.4)	21 (16.5)	18 (13.2)	14 (10.5)	
Overweight n (%)		21 (4.9)	4 (3.1)	10 (7.4)	7 (5.3)	
Obese n (%)		7 (1.6)	3 (2.4)	0 (0)	4 (3.0)	
Parental education level n (%)	351					0.468
Elementary/vocational school		138 (39.3)	45 (40.5)	51 (42.5)	42 (35.0)	
College/university		213 (60.7)	66 (59.5)	69 (57.5)	78 (65.0)	
Family income in NOK^c^ n (%)	348					0.421
<200,000–749,999		74 (21.3)	21 (19.1)	21 (17.6)	32 (26.9)	
750,000–1,249,999		178 (51.1)	57 (51.8)	66 (55.5)	55 (46.2)	
1,250,000- > 2,000,000		96 (27.6)	32 (29.1)	32 (26.9)	32 (26.9)	
Immigrant^d^ n (%)	351	8 (2.3)	2 (1.8)	2 (1.7)	4 (3.3)	0.633
Fish oil supplements^e^ (n (%))	423					0.719
Never		228 (53.9)	66 (48.2)	81 (55.5)	81 (57.9)	
1–3 times/month		54 (12.8)	18 (13.1)	19 (13.0)	17 (12.1)	
1–3 times/week		47 (11.1)	21 (15.3)	14 (9.6)	12 (8.6)	
4–6 times/week		21 (5.0)	7 (5.1)	6 (4.1)	8 (5.7)	
Every day		73 (17.3)	25 (18.2)	26 (17.8)	22 (15.7)	

Data are given as mean ± SD or n (%). *Abbreviations*: *SD* Standard deviation, *NOK* Norwegian kroner

^a^ One-way ANOVA test (continuous variables) and Pearson’s Chi-square test (*X* ^2^) (categorical variables) for comparison between treatment groups

^b^Cole’s age and sex-specific BMI cut off points for underweight [25], and overweight and obesity [26] for adolescents age 14.5 years

^c^ 100 NOK = approximately 10€/11$

^d^ Immigrant was defined as participants who’s both parents and themselves were born outside Norway

^e^ N (%) of participants reporting to consume fish oil as dietary supplements

### Effects of the intervention on the d2 test of attention

All groups showed significant improvement post compared to pre intervention in concentration performance (CP), total performance (TN) and processing speed (TN-E) (Table 3). The mean improvement in TN was significantly lower in the meat and supplement group compared to the fish group when controlling for baseline level. The supplement group also showed significant lower improvement in TN-E compared to the fish group when controlling for baseline. The results were only marginally affected when adjusting for dietary compliance. No association between the intervention and CP was observed (Table 3).

**Table 3:** Predicted change in d2 outcomes after intervention with fish (*n* = 137), meat (*n* = 148), n-3 supplements (*n* = 141)

					Models adjusted for:			
		Crude			Baseline score^*a*^		Baseline, dietary compliance^*b*^	
d2 test of attention outcomes^c^		Pre Mean ± SD	Post Mean ± SD	*P-within* ^*d*^	Coefficients (95% CI)	*P-value*	Coefficients (95% CI)	*P-value*
Concentration performance (CP)								
	Fish	142.4 ± 35.0	177.3 ± 36.8	<0.001	1 (ref.)		1 (ref.)	
	Meat	146.3 ± 31.5	178.5 ± 37.6	<0.001	−2.3 (−6.8, 2.2)	0.317	−3.4 (−8.2, 1.3)	0.159
	Supplement	143.8 ± 35.8	176.1 ± 40.7	<0.001	−2.4 (−6.9, 2.2)	0.306	−4.4 (−9.7, 1.0)	0.110
Total performance (TN-E)								
	Fish	377.4 ± 73.5	453.3 ± 72.6	<0.001	1 (ref.)		1 (ref.)	
	Meat	379.1 ± 73.2	446.3 ± 77.0	<0.001	−7.9 (−17.4, 1.6)	0.103	−10.0 (−20.1, 0.0)	0.051
	Supplement	381.9 ± 77.4	446.5 ± 83.2	<0.001	−10.4 (−20.0, −0.7)	0.035	−14.1 (−25.5, −2.7)	0.015
Processing speed (TN)								
	Fish	408.7 ± 80.3	482.1 ± 79.0	<0.001	1 (ref.)		1 (ref.)	
	Meat	404.0 ± 79.6	466.1 ± 80.4	<0.001	−11.8 (−23.3, −0.4)	0.042	−13.3 (−25.5, −1.2)	0.031
	Supplement	413.9 ± 83.9	472.8 ± 89.1	<0.001	−13.4 (−24.9, −1.8)	0.024	−16.0 (−29.6, −2.4)	0.022

Pre and post data are presented as mean ± SD and difference between treatment groups presented as coefficients (95% CI). Abbreviations: *SD* Standard deviation, *CI* Confidence interval

^a^Between group differences analyzed using linear mixed effects model, with school class as random intercept

^b^Adjusted for the equivalent outcome at baseline and for dietary compliance (i.e. the total intake of study meals or supplements) during the trial

^c^An increase in d2 outcomes indicates improvement

^d^Paired-samples T-test for comparison within treatment groups from pre to post intervention

Mean commission errors (E2) decreased in all groups at post compared to pre intervention, while omission errors (E1) did not (Table 4). The IRR of E1 errors decreased in the meat group compared to the fish group when controlling for baseline level. However, the difference was no longer significant when controlling for dietary compliance. No significant differences between the intervention groups were seen in E2 or total errors during the trial (Table 4).

**Table 4:** Predicted change in d2 outcomes after intervention with fish (*n* = 137), meat (*n* = 148), n-3 supplements (*n* = 141)

					Models adjusted for:			
		Crude			Baseline score^*a*^		Baseline, dietary compliance^*b*^	
d2 test of attention outcomes^c^		Pre Mean ± SD	Post Mean ± SD	*P-within* ^*d*^	IRR (95% CI)	*P-value*	IRR (95% CI)	*P-value*
E1 errors								
	Fish	24.6 ± 28.3	25.3 ± 27.9	0.544	1 (ref.)		1 (ref.)	
	Meat	19.0 ± 19.3	16.4 ± 15.9	0.074	0.85 (0.74, 0.98)	0.026	0.88 (0.76, 1.02)	0.084
	Supplement	25.4 ± 23.0	22.8 ± 24.1	0.161	1.01 (0.83, 1.23)	0.933	1.06 (0.88, 1.29)	0.528
E2 errors								
	Fish	6.7 ± 8.8	3.5 ± 6.1	<0.001	1 (ref.)		1 (ref.)	
	Meat	5.9 ± 6.2	3.5 ± 9.2	0.001	0.91 (0.59, 1.39)	0.648	0.92 (0.60, 1.40)	0.681
	Supplement	6.6 ± 9.0	3.6 ± 6.3	<0.001	0.88 (0.63, 1.24)	0.469	0.90 (0.61, 1.32)	0.586
Total errors								
	Fish	31.3 ± 32.6	28.8 ± 30.1	0.093	1 (ref.)		1 (ref.)	
	Meat	24.9 ± 21.7	19.9 ± 20.8	0.006	0.88 (0.75, 1.02)	0.094	0.91 (0.77, 1.07)	0.247
	Supplement	32.0 ± 27.6	26.4 ± 27.1	0.004	0.96 (0.80, 1.15)	0.671	1.03 (0.86, 1.23)	0.772

Pre and post data are presented as mean ± SD and difference between treatment groups presented as coefficients (95% CI). Abbreviations: *SD* Standard deviation, *IRR* Incidence rate ratio, *CI* Confidence interval, *E1* Errors of omission, *E2* Errors of commission; Total errors (E1 + E2 errors)

^a^Between group differences analyzed using general estimates equation model, with the negative binomial distribution, exchangeable correlation structure and robust standard errors

^b^Adjusted for the equivalent outcome at baseline and for dietary dietary compliance (i.e. the total intake of study meals or supplements) during the trial

^c^A decrease in the number of errors indicates improvement

^d^Paired-samples T-test for comparison within treatment groups from pre to post intervention

Additional analyzes with further adjustments for parental education level, at home use of omega-3 supplements and fatty fish intake (dinner and bread spread) at baseline did not affect the results. There were no interaction effects between dietary compliance and the intervention in neither of the models (not shown).

**Fig. 2:**
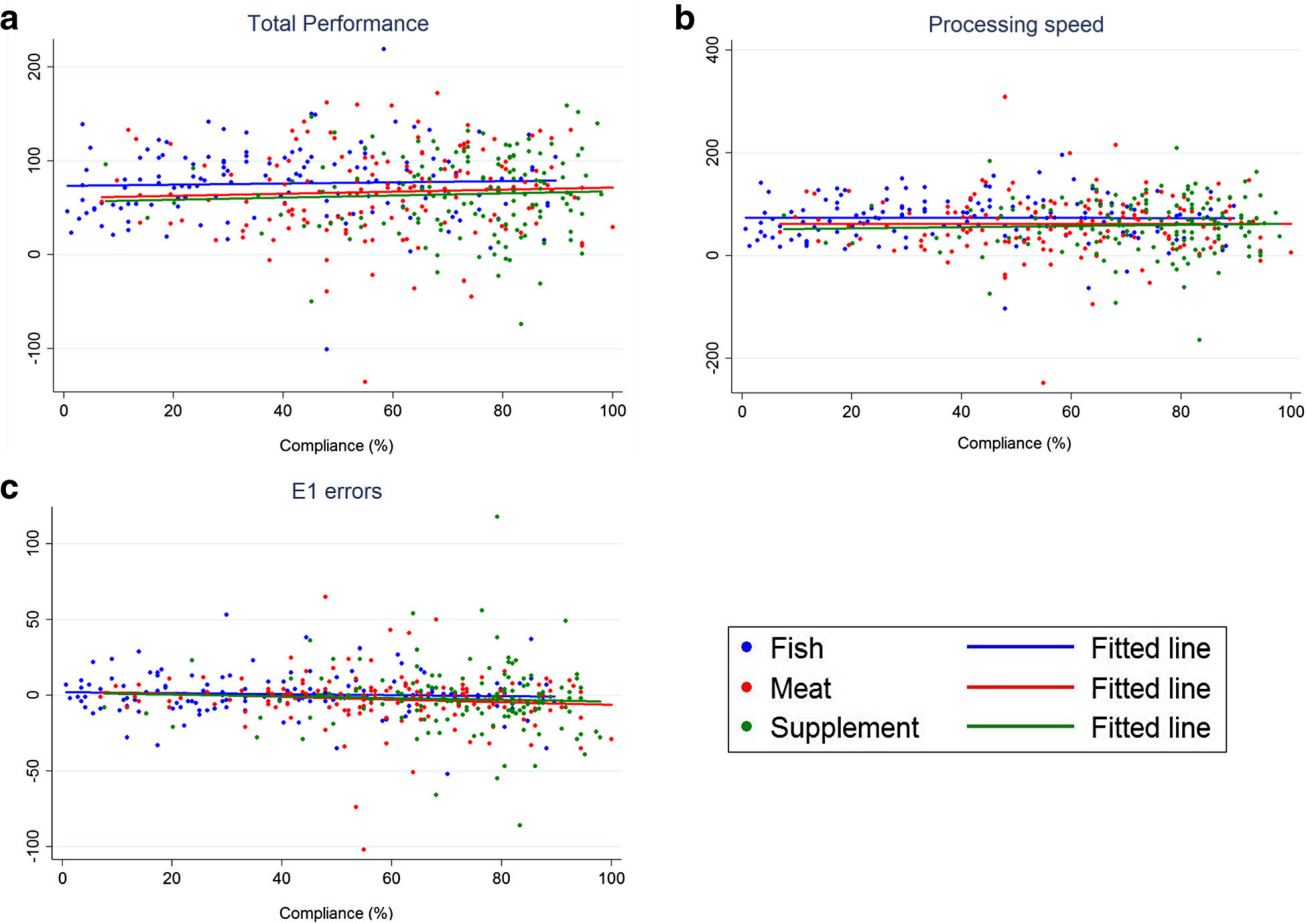
Associations between dietary dietary compliance (the total intake of study meals or supplements, given in %) and the change (post-pre) in: **a**) total performance (TN-E), **b**) processing speed (TN) and **c**) E1 errors (Omission errors) in the d2 test of attention, given for the fish, meat and supplement group. Crude fitted regression line for each intervention group

There were no significant correlations between dietary compliance and the change (post-pre) in TN, TN-E and E1 errors in neither of the intervention groups, as shown in Fig. 2.

### Dietary compliance and background diet

As described in Skotheim et al. [19], dietary compliance records showed a significantly lower total intake in the fish group compared to the meat and the supplement group. The percentage of participants who consumed at least half of the fish/meat/capsules during the trial was 38%, 66% and 87% in the fish, meat and supplement group, respectively [19].

There were no significant differences between groups in their reported habitual intake of fish (as bread spread or dinner), red or white meat (for dinner) over the course of the intervention (Additional file 1: Table S1). At baseline, the mean intake of fish for dinner was 1.5 ± 1.0 meals per week, of which 1.0 ± 1.0 meals were fatty fish. During the trial, the reported habitual intake of fish as bread spread for lunch and fatty fish for dinner decreased within all treatment groups, but no differences were observed between the groups (Table S1). The percentage of participants who responded “no” to the overall question regarding whether they used fish oil supplements at home increased significantly within all groups during the trial, to 69%, 74% and 69% in the fish, meat and supplement group, respectively, but no differences between groups were observed.

## Discussion

The results suggest that although all groups improved significantly in processing speed (TN) and total performance (TN-E), the mean improvements were 8–13 characters less in the meat- and supplement groups compared to the fish group. This corresponds to approximately 2% of the total 658 characters in the test. The risk of omission (E1) errors decreased in the meat group compared to fish in the baseline-adjusted analyses, but not when additionally adjusting for dietary compliance.

A previous RCT in 8–11 year old children in Denmark used the d2 test of attention to investigate the effects of providing school meals based on the New Nordic diet. The intervention lasted for 3 months and included fish approximately once per week. Contrary to our results, no effects were observed on neither TN nor TN-E compared to the control group. They found that the percentages of E1 and E2 errors were higher after the intervention compared to the control period, when the children ate their usual packed lunch [16]. However, this is not in accordance with the decrease in E1 errors that we found in the meat group in the present study. Our findings in TN (and TN-E, which seems to be driven by TN) suggests that there could be a link between fish intake and the speed of processing information. To our best of knowledge, no other RCTs have examined the effects of fish intake on attention or processing speed in healthy young subjects. However, although not directly comparable to our sample of adolescents, two cross-sectional studies in middle-aged adults (45–70 y, *n* = 1613) [27] and elderly (70-74y, *n* = 2031) [28] reported positive associations between fish consumption and cognitive speed, motor speed and attention. Some studies have also been conducted in adolescents, using other cognitive outcomes than attention or processing speed. Kim et al. [14] found a positive association between the reported fish intake and average school grades in Swedish adolescents (15 y, *n* = 9448). The association seemed to be dose-dependent between the number of times the respondent reported to consume fish [14]. Åberg et al. [13] used questionnaire data from male respondents in the same study and linked them with records on intelligence test performance at age 18, collected at the Swedish Military Conscription Register (*n* = 3972). These data showed that fish consumption at age 15 was positively associated with higher performances in the domains ‘combined intelligence’, ‘verbal performance’ and ‘visuospatial performance’ at age 18, irrespective of educational level [13]. Finally, De Groot et al. [15] found no association between fish consumption and attention *problems*, which cannot be directly compared to the d2 test of attention measure, in Dutch adolescents (12–18 y, *n* = 700). However, they found an inverted u-shape association between fish intake and more advanced vocabulary and higher end term grades, showing that eating fish was beneficial, but eating more fish than recommended (defined as ~450 mg/d EPA + DHA) no longer seemed to be effective [15].

We did not have a pure placebo group, and thus, it is not possible to separate the effects of the intervention from a learning effect that may have occurred from conducting the same test twice. However, since a learning effect should be equally distributed in the groups, the effect of one intervention over the other(s) should be detectable when comparing the groups against each other. Our results are difficult to interpret, because the beneficial effect in the fish group on TN and TN-E was observed even among those with very low dietary compliance. This makes it difficult to conclude on the relationship between fish intake and performance in TN and TN-E. The significant decrease of omission (E1) errors in the meat group compared to fish in the crude analyses is also inconclusive, because it disappeared when adjusting for dietary compliance.

There were no effects on the d2 test in the supplement group which is interesting, because of the relatively good dietary compliance in this group, and the documented roles of n-3 LCPUFAs in brain development and function [6]. Previous studies also report inconsistent effects of supplementing with n-3 LCPUFAs on cognitive outcomes in healthy school-aged children [11, 12]. As stated in the meta-analysis by Mazereeuw et al., the contradictory findings in RCTs with n-3 LCPUFA supplements could be due to methodical heterogeneities in the dose, sample size, duration, or the cognitive tests used [10]. The dose of n-3 LCPUFAs used in the present study was smaller compared to most of the previous studies with supplements, because of our aim to keep the dose equal in the fish and supplement group. The superior effect of fish meals compared to the n-3 LCPUFA supplements could also be due to differences in bioavailability. The n-3 LCPUFAs in fatty fish are bound to phospholipids and triacylglycerol in a 40:60 ratio, whereas n-3 LCPUFAs in most supplements are usually exclusively bound to triacylglycerol, which is assumed to be less bioavailable [29]. As mentioned in the introduction, it could be that the discrepancy between the results in the fish and supplement group in this study were due to additional nutrients, or the combination of nutrients found in fatty fish and not n-3 LCPUFAs alone.

Our results should give implications for future research by shifting the focus from the roles of single nutrients to the roles of food and dietary patterns, reflecting the complexity of diet and eating behavior. These results emphasize the importance of monitoring adherence to protocol in dietary intervention trials, as the records clearly show that interventions with pills or supplements are less demanding for participants compared to interventions with food. The adolescents’ habitual diet was low in fish [30], which probably increased the importance of quality and tastiness of the meals even more to ensure acceptable compliance. We had to serve the meals cold due to practical causes, which could be the main reason for the low dietary compliance observed in the fish group. It should be discussed further whether the RCT design is the most appropriate when studying the effects of diet and intake of foods. Perhaps a prospective cohort study using validated dietary assessment tools and school derived grade-levels as a generalizable outcome could be more appropriate to investigate the relationship between diet and cognition in healthy school children- and adolescents.

The main limitations with the present study are, as previously mentioned, the low dietary compliance particularly observed in the fish group, the lack of a placebo group and the ceiling effect in the reading and spelling test “Kartleggeren”, which left us with only one cognitive outcome. In addition, the duration of 12 weeks might have been too short, but in practice, it is difficult to conduct this type of study for very much longer due to how the schools are organized with several holidays during the year. Even though the study sample was large and derived from nearly all boroughs, generalizability is compromised by the restricted area of Bergen (277,000 inhabitants). In addition, the proportion of university/college education was higher among the responding caregivers than in the general Norwegian population of 30–59 years [31], however adjusting for parental education did not affect the results. It was not possible to blind the participants in this trial, but it was communicated that the aim was to investigate school meals in general and not fish, as an attempt to avoid performance bias. Although the recording of dietary compliance is a strength, it is limited by the fact that it was estimated by eye rather than using an objective measure such as weighing out food portions. An important strength is that we succeeded to administer the cognitive tests at the same time of day, and with the same researchers at each school, which increases internal validity [32]. The registration of dietary compliance is also an important strength. In addition, as the aim was to include a representable sample of adolescents, the study did not exclude those with cognitive disorders or those who already took n-3 supplements.

## Conclusions

We observed a small beneficial effect of fatty fish, compared to meat meals and supplements on processing speed compared to intakes of similar meals with meat or n-3 LCPUFA supplements. However, the group differences were small and difficult to interpret due to low dietary compliance. Thus, more studies are needed to understand the implication of our results. This study shows that different taste preferences among participants is challenging in intervention trials with food. A prospective cohort design may be a better alternative when studying diet and cognitive outcomes in the future.

## Additional file

Additional file 1: Table S1. Changes within and between treatments in reported meals per week (besides the intervention) of background diet from pre to post. Data given as mean ± SD or mean (95% CI). *n* = 137 in fish group, *n* = 148 in meat group and *n* = 141 in supplement group. (DOCX 18 kb)

## Abbreviations

CI: Confidence interval
CP: Concentration performance
DHA: Docosahexaenoic acid
E total: Total errors
E1: Errors of omission
E2: Errors of commission
EPA: Eicosapentaenoic acid
FFQ: Food frequency questionnaire
FINS: Fish intervention studies
GEE: Generalized estimates equation
IRR: Incidence rate ratio
LCPUFA: Long-chain polyunsaturated fatty acids
n-3: Omega-3
RCT: Randomized controlled trial
SD: Standard deviation
TN: Processing speed
TN-E: Total performance
