# Supplementary material for: Fatty fish intake and attention performance in 14–15 year old adolescents: FINS-TEENS - a randomized controlled trial
Source: Nutr J. 2017 Oct 2;16:64. doi: 10.1186/s12937-017-0287-9 (PMC5625698; doi:10.1186/s12937-017-0287-9)
Supplement: Additional file 1: Table S1. — Changes within and between treatments in reported meals per week (besides the intervention) of background diet from pre to post. Data given as mean ± SD or mean (95% CI). n = 137 in fish group, n = 148 in meat group and n = 141 in supplement group. (DOCX 18 kb) [file 12937_2017_287_MOESM1_ESM.docx]

**Table S1** Changes within and between treatments in reported meals per week (besides the intervention) of background diet from pre to post. Data given as mean ± SD or mean (95% CI). n=137 in fish group, n=148 in meat group and n=141 in supplement group.

| Variables | | Pre  Mean ± SD | Post  Mean ± SD | Change  Mean (95% CI) | *P-value^1^* | *P-value^2^* |
| --- | --- | --- | --- | --- | --- | --- |
|  | | |  |  |  |  |
| Fish for dinner | | |  |  |  | 0.758 |
|  | Fish | 1.49 ± 0.99 | 1.48 ± 0.95 | -0.01 (-0.14, 0.14) | 0.976 |  |
|  | Meat | 1.42 ± 0.90 | 1.35 ± 0.94 | -0.07 (-0.22, 0.07) | 0.313 |  |
|  | Supplement | 1.48 ± 0.88 | 1.42 ± 0.93 | -0.07 (-0.23, 0.09) | 0.397 |  |
|  |  |  |  |  |  |  |
| Herring/mackerel/salmon for dinner | | |  |  |  | 0.580 |
|  | Fish | 0.97 ± 0.96 | 0.73 ± 0.63 | -0.24 (-0.40, -0.08) | 0.003 |  |
|  | Meat | 0.93 ± 0.87 | 0.71 ± 0.80 | -0.21 (-0.33, -0.09) | 0.001 |  |
|  | Supplement | 1.00 ± 1.04 | 0.68 ± 0.74 | -0.32 (-0.47, -0.16) | <0.001 |  |
|  |  |  |  |  |  |  |
| Fish as bread spread | | |  |  |  | 0.884 |
|  | Fish | 0.68 ± 1.04 | 0.54 ± 0.86 | -0.14 (-0.29, 0.01) | 0.062 |  |
|  | Meat | 0.64 ± 1.03 | 0.44 ± 0.77 | -0.20 (-0.36, -0.03) | 0.019 |  |
|  | Supplement | 0.57 ± 0.93 | 0.40 ± 0.69 | -0.17 (-0.29, -0.04) | 0.008 |  |
|  |  |  |  |  |  |  |
| Red meat for dinner | |  |  |  |  | 0.547 |
|  | Fish | 2.08 ± 1.07 | 2.11 ± 1.07 | 0.03 (-0.18, 0.24) | 0.772 |  |
|  | Meat | 2.11 ± 1.12 | 2.21 ± 1.09 | 0.10 (-0.08, 0.29) | 0.268 |  |
|  | Supplement | 2.13 ± 1.03 | 2.08 ± 1.09 | -0.05 (-0.24, 0.15) | 0.615 |  |
|  |  |  |  |  |  |  |
| White meat for dinner | | |  |  |  | 0.755 |
|  | Fish | 1.39 ± 0.96 | 1.43 ± 0.92 | 0.05 (-0.14, 0.24) | 0.628 |  |
|  | Meat | 1.28 ± 0.92 | 1.35 ± 0.89 | 0.07 (-0.09, 0.23) | 0.392 |  |
|  | Supplement | 1.40 ± 0.94 | 1.39 ± 0.99 | -0.02 (-0.16, 0.13) | 0.826 |  |

Abbreviations: CI, confidence interval; SD, standard deviation.

^1^ Paired-samples T-test for comparison within treatment groups from baseline to end.

^2^ One-way ANOVA test for differences between treatment groups.
